# Assessing affect in adolescents with e-diaries: multilevel confirmatory factor analyses of different factor models

**DOI:** 10.3389/fpsyg.2023.1061229

**Published:** 2023-06-23

**Authors:** Matthias F. Limberger, Florian Schmiedek, Philip S. Santangelo, Markus Reichert, Lena M. Wieland, Oksana Berhe, Andreas Meyer-Lindenberg, Heike Tost, Ulrich W. Ebner-Priemer

**Affiliations:** ^1^Mental mHealth Lab, Institute for Sport and Sport Science, Faculty for Humanities and Social Sciences, Karlsruhe Institute of Technology (KIT), Karlsruhe, Germany; ^2^Leibniz Institute for Research and Information in Education (DIPF), Frankfurt, Germany; ^3^IDeA Research Center for Individual Development and Adaptive Education of Children at Risk, Frankfurt, Germany; ^4^Department of Educational Psychology, Goethe University Frankfurt, Frankfurt, Germany; ^5^Behavioural Health Technology Interventions, Department of Behavioural and Cognitive Sciences, Faculty of Humanities, Education and Social Sciences, Université du Luxembourg, Esch-sur-Alzette, Luxembourg; ^6^Department of Psychiatry and Psychotherapy, Central Institute of Mental Health, Medical Faculty Mannheim, Heidelberg University, Mannheim, Germany; ^7^Department of eHealth and Sports Analytics, Faculty of Sports Science, Ruhr University Bochum, Bochum, Germany; ^8^Department of Psychiatry and Psychotherapy, Central Institute of Mental Health, Medical Faculty Mannheim, University of Heidelberg, Mannheim, Germany

**Keywords:** psychometric properties, affect, adolescents, reliability, ambulatory assessment

## Abstract

In the last two decades, e-diary studies have gained increasing interest, with a dominant focus on mood and affect. Although requested in current guidelines, psychometric properties are rarely reported, and methodological investigations of factor structure, model fit, and the reliability of mood and affect assessment are limited. We used a seven-day e-diary dataset of 189 adolescent participants (12–17  years). The e-diary affect assessments revealed a considerable portion of within-person variance. The six-factor model showed the best model fit compared to the less complex models. Factor loadings also improved with the complexity of the models. Accordingly, we recommend that future e-diary studies of adolescents use the six-factor model of affect as well as reporting psychometric properties and model fit. For future e-diary scale development, we recommend using a minimum of three items per scale to enable the use of confirmatory multilevel factor analyses.

## 1. Introduction

In the last two decades, e-diary studies have gained increasing interest (Hamaker and Wichers, 2017) with a dominant focus on mood and affect. This increasing interest is likely fueled by the possibility of assessing psychological phenomena in real time and in everyday life (Trull and Ebner-Priemer, 2013). According to recent considerations, these two features increase ecological validity and decrease recall bias (Conner and Barrett, 2012). Another prominent feature of e-diary assessments is the possibility of repeatedly assessing phenomena of interest over time, which enables researchers to reveal within-person dynamics (Ebner-Priemer and Trull, 2012; Hamaker, 2012; Trull et al., 2015). It is important to acknowledge that findings observed at the within-person versus between-person level can differ substantially. An impressive example of such differences is provided by Kamarck et al. (2003), who showed that at a within-person level, blood pressure is negatively related to physical activity, whereas at the between-person level, a positive relation is evident (i.e., blood pressure increases with physical activity, whereas well-trained physically active individuals tend to have lower blood pressure). Accordingly, between- and within-person levels of analysis must be clearly differentiated and cannot substitute for each other (Molenaar, 2004; Nesselroade, 2004).

Importantly, Wilhelm and Schoebi (2007) showed that the factor structure of certain psychological phenomena, such as affect and mood, can also differ at the within- and between-person levels. This implies that when addressing within-person research questions, we must collect and analyze our data on a within-person level, including model fit and psychometric properties. This is in accordance with recent guidelines (Trull and Ebner-Priemer, 2020), but investigations of research practice reveal that only 30% of e-diary studies report psychometric properties on a within-person level (Trull and Ebner-Priemer, 2020). Although disappointing, this is not surprising, as scales of affect or mood that are validated on a within-person level are scarce. To our knowledge, there is one published methodological study (Wilhelm and Schoebi, 2007) and one meta-analysis (Scott et al., 2020) of adults and one published methodological study of children (Leonhardt et al., 2015) of the factor structure and psychometric properties of e-diary affect and mood scales. Studies of adolescents are missing completely. In the following, before stating our research hypotheses, we briefly introduce different dimensional models of affect that have been discussed in the literature and report how psychometric properties and model fit can be estimated on a within-person level.

Not surprisingly, the history of psychological models of affect and mood, as well as that of approaches to assessing these constructs, are long and controversial. One of the earliest conceptualizations was introduced by Wundt (1896), who located subjective feeling states within a three-dimensional space, defined by the bipolar dimensions of valence (positive–negative), arousal (calm – excited), and tension (tense – relaxed) (see Scherer, 2005). In the following decades, researchers experienced difficulties in distinguishing Wundt’s arousal and tension dimensions, and at least over the last two decades, two-dimensional approaches have dominated the discussion about the structure of mood and affect. Russell (1980) proposed a circumplex model of mood and affect representing core affect with the two dimensions of valence and arousal, with these two dimensions assumed to be independent. Thayer (1998) assumed that tense arousal (relaxation-tension) and energetic arousal (tiredness–wakefulness) have to be distinguished as two basic arousal dimensions. The dimensions in the Thayer model can be viewed as a 45-degree rotation of Russel’s model. Thayer (1998) provided a unipolar adjective checklist to assess energetic and tense arousal.

Positive and negative affect as two uncorrelated basic dimensions of affect were proposed by Watson and Tellegen (1985). The authors developed the Positive and Negative Affect Schedule (PANAS) with ten unipolar items for each dimension [positive and negative affect (Watson et al., 1988)]. In the current debate, the two orthogonal dimension models are still widely accepted and most often used, although it has been repeatedly demonstrated that the three-dimensional structure cannot be reduced to a two-dimensional model without losing information (Matthews et al., 1990; Schimmack, 1999; Schimmack and Grob, 2000; Schimmack and Reisenzein, 2002). To examine different models of affect, we used a) the scales of positive and negative affect in a two-factor model; b) the pleasure-arousal model with the four factors of *low arousal*, *high arousal*, *pleasure*, and *displeasure*; and c) a model with six factors (*tension*, *tiredness*, *alertness*, *calmness*, *good mood,* and *bad mood*) to display the three-dimensional model. As shown by Brose et al. (2015) differences in affect on between-person level could not directly be transferred to variation at the within-person level. All models mentioned above are developed on basis of the between-person level, so differences in the affect structure on the within-person level have to be expected.

To empirically compare different affect and mood models, psychometric properties, such as reliability, factor structure, and model fit, are usually evaluated. Given the multilevel structure of experience sampling data and substantive interest in relations at both the between- and within-person levels of analysis, reliability indices should be estimated within a multilevel model comprising the between- and within-person levels (Ebner-Priemer et al., 2020). Geldhof et al. (2014) have shown how to estimate McDonald’s Omega reliability separately at the within- and between-person levels within a common multilevel confirmatory factor analysis (MCFA) and empirically demonstrated that this yields more precise estimates of reliability than Cronbach’s alpha (even when calculated in a CFA). A benefit of such an approach based on a CFA model is that it allows for heterogeneous relations between indicators and their underlying common factor(s) (Geldhof et al., 2014). In practice, this denotes that not all items must have the same factor loadings on a given scale, as implied by Cronbach’s alpha. Unfortunately, psychometric properties of e-diary affect scales and item sets are rarely reported (Brose et al., 2020; Trull and Ebner-Priemer, 2020). When psychometric properties are reported, different indices were used, and these are largely not capable of appropriately taking within-person variance into account [for exceptions, see (Buse and Pawlik, 1996; Zelenski and Larsen, 2000; Buse and Pawlik, 2001; Schimmack, 2003; Cranford et al., 2006; Wilhelm and Schoebi, 2007)].

Another benefit of using MCFA models to evaluate psychometric properties is that model fit indices are provided automatically. In simple terms, model fit indices describe how well the multilevel data fit the proposed model or if the specified MCFA is appropriate for the given data. This allows testing, for example, whether the underlying factor structure of a given set of mood items better fits a two- or a three-dimensional model. Various well-established measures exist for this purpose, such as the comparative fit index (CFI), root mean square error of approximation (RMSEA), and standardized root mean square residual (SRMR). In addition, the Satorra-Bentler scaled χ^2^-difference test (Satorra and Bentler, 2001) can be used to compare different models and test the superiority of a model. Unfortunately, reports of model fit of the dimensional models used here are rare. The only exception in the area of e-diaries and affect, to our knowledge, is the study by Leonhardt et al. (2015), who tested different models and described their model fit for a population of children. Investigating affect over five consecutive days in 214 children (age 8–11 years), Leonhardt et al. (2015) revealed that a six-factor model (representing the three dimensions good-bad mood, alertness-tiredness, calmness-tension), did better in describing affect in children, compared to a two-factor model (representing the positive–negative affect dimension), and compared to a four-factor model (representing the two dimensions pleasure and arousal). To assess the momentary affective state, we used the 20 affective adjectives: *cheerful, stressed, interested, content, pleasant, afraid, on edge, good, exhausted, mad, delighted, active, faint, fantastic, unhappy, concentrated, rested, miserable, tired* and *anxious* which were used by (Leonhardt et al., 2015) in children to examine the model fit of different factor models.

Adolescence is an episode of change and moodiness. Besides physical conditions, the kind of social interaction as well as cognitive processing changes (Graber and Brooks-Gunn, 1996). There is considerable evidence that adolescents experience more extreme affect (both positive and negative) on average, as well as more variable mood states in their everyday lives than adults (Larson et al., 1980). Many adolescents widen their social network by establishing more types of relationships than in childhood (Cole et al., 2004). The development of the limbic system, responsible for emotional processing (Dahl, 2004), is faster than that of the frontal lobe, which is responsible for judgment and inhibition (Fellows and Farah, 2007). In this development period, heightened emotional distress is also a characteristic (Ibraheim et al., 2017). For these reasons, adolescents exhibit greater difficulties in emotion dysregulation when compared to adults and children (Silvers et al., 2012).

## 2. Method

### 2.1. Hypotheses

To increase knowledge of psychometric properties and the fit of different model of affect in e-diary assessments of adolescents, we used a dataset of 189 adolescent participants between 12 and 17 years of age (Reichert et al., 2020) and compared the data to the results of the children sample reported in Leonhardt et al. (2015). To increase comparability, we retained to the analyses by Leonhardt et al. (2015), comparing two, four, and six-factor models of affect.

Based on prior research of affect dynamics, we expected our repeated momentary measures of affective states to reveal a considerable portion of within-person variance across all affect items shown in the ICCs (of 60% or more), (Hypothesis 1a) of comparable size to the assessment of affect in children (Leonhardt et al., 2015), and ISDs higher than that of the child Sample (Hypothesis 1b). Analogous to the child sample reported in Leonhardt et al. (2015), we hypothesized that in our adolescent sample, the six-factor model of affect would show a better model fit than the two-factor or pleasure-arousal model (Hypothesis 2). We expected an overall pattern of descriptively higher factor loadings on a within- and between-level in the six-factor model compared to patterns of the other two simpler models. In addition, we expected our repeated momentary reports of affective states to add up to reliable affect factors at the between- and within-person levels in the six-factor model (Hypothesis 3).

### 2.2. Participants

A total of 189 adolescent participants between 12 and 17 years of age were enrolled in the study. Participants were recruited through the Impact of Urbanicity on Genetics, Cerebral Functioning and Structure and Condition in Young People (URGENCY Study (Reichert et al., 2020)) conducted at the Psychiatric-Epidemiological Center of the Central Institute of Mental Health in Mannheim, Germany from December 2014 to January 2017. Participants with acute diseases, mental disorders, cardiovascular disorders, or chronic endocrine or immunological diseases were excluded from the study. Participants carried an e-diary for seven consecutive days while undergoing their everyday life activities. For detailed information on the recruitment and methodological approaches used, see (Reichert et al., 2017). Three participants were excluded from the analyses due to low adherence to the e-diary protocol (< 30% answered prompts) as recommended by current guidelines (Trull and Ebner-Priemer, 2020). The final sample consisted of *N* = 186 participants (52% male, 48% female) with a mean age of 15.02 years (*SD* = 1.70).

Details on the characteristics and sampling procedure (daily assessments conducted once every morning over one school week, i.e., five consecutive days) of the child sample to which we compare our adolescent sample are described in Leonhardt et al. (2015). Most importantly, in both samples, identical adjectives were used to assess momentary affective states.

### 2.3. Ambulatory assessment procedure

Participants received a study smartphone (Motorola Moto G) programmed with movisensXS (movisens GmbH, Karlsruhe). All participants were thoroughly instructed and trained on the use of the e-diary. Participants repeatedly reported their momentary affect in real time *via* ratings entered into the e-diaries for the following 7 days. All items were presented on seven-point rating scales with reversed polarity and in mixed order.

The e-diary sampling scheme involved a combination of fixed time points, random time points, and GPS-triggered assessments to increase the likelihood of assessment in different environments. Fixed time-based triggers prompted participants at 16:30 and 20:20 on weekdays and at 9:30 and 19:50 on weekends to rate their momentary affective state. Moreover, participants were repeatedly prompted between 16:00 and 20:30 on weekdays (to not disturb students during school hours) and between 9:00 and 20:00 on weekends. The additional GPS-based trigger prompted participants to rate their affective state whenever they moved over a distance of 0.5 km or more. Prompts were programmed to occur with a minimum interval between two prompts of 37 min and a maximum interval of 77 min. This sampling scheme resulted in four to seven prompts per day on weekdays and eight to 17 prompts per day on weekends. Participants were asked to respond to the alarm immediately whenever possible, but they could also delay responding for up to 15 min. The e-diary automatically time-stamped each response. After completing the 7 days of assessment, participants returned the e-diaries, were debriefed, and were financially compensated. The study protocol was approved by the Ethical Committee of the Medical Faculty of Heidelberg University, Germany, and was carried out following the Declaration of Helsinki (World Medical Association, 2013). All participants, as well as their legal guardians, provided written informed consent prior to their inclusion in the study.

### 2.4. Data analyses

In the first step, we calculated the intraclass correlation coefficient (ICC) of all affect items to examine the ratio of between- and within-person variance (Singer and Willett, 2003). We also computed the intraindividual standard deviation (ISD) of each item (Eid and Diener, 1999) to capture variability around participants’ individual means across all measurement occasions. ISDs allow for comparing variability across items, with higher ISDs indicating higher variability. In addition, to examine affective instability, we calculated the mean square successive differences (MSSD) (Neumann et al., 1941; Ebner-Priemer et al., 2007), which takes into account the change in affect between one measurement point and the next, and is therefore an important index in e-diary studies, especially for the description of stability of affect. Since e-diary studies can often only use a small selection of affect items, this information seems to us to be an important criterion for selecting appropriate items to measure affect instability.

We compared our sample of adolescents with the child sample of Leonhardt et al. (2015) to examine the increased variation and instability in affect (e.g., Larson et al., 1980; Cole et al., 2004). For the analyses, which directly compare the child sample of Leonhardt et al. (2015) to our sample (Table 1), we adapted the scale by transforming the values from 1–7 to 1–5 (using formula ((x-1)/6)*4 + 1). We performed t-tests, to test the differences in mean and ISD values between children and the range-adjusted values of the adolescents (ranging from 1 to 5).

**Table 1:** Item means, ISDs, MSSDs, and ICCs of the present Sample of adolescents and the children sample reported in Leonhardt et al. (2015).

Item	Adolescents (1–7)				Children (1–5)			Adolescents (1–5)	
	Mean (SD)	Average ISD (SD)	MSSD (SD)	ICC	Mean (SD)	Average ISD (SD)	ICC	Mean (SD)	Average ISD (SD)
cheerful	5.21 (1.45)	1.06 (0.44)	1.66 (1.38)	0.39	4.15 (0.78)	0.72 (0.57)	0.34	3.75 (0.97)	0.70 (0.29)
content	4.98 (1.47)	1.14 (0.46)	2.23 (1.86)	0.31	4.08 (0.79)	0.75 (0.56)	0.34	3.65 (0.98)	0.76 (0.31)
good	5.05 (1.43)	1,11 (0.47)	2.19 (3.09)	0.31	4.23 (0.78)	0.70 (0.56)	0.36	3.70 (0.96)	0.74 (0.31)
delighted	4.74 (1.56)	1.19 (0.48)	2.39 (2.06)	0.33	3.85 (0.87)	0.86 (0.56)	0.32	3.49 (1.04)	0.79 (0.32)
fantastic	4.75 (1.53)	1.12 (0.50)	2.18 (2.14)	0.37	3.61 (1.07)	0.77 (0.52)	0.53	3.50 (1.02)	0.75 (0.33)
pleasant	4.81 (1.52)	1.19 (0.45)	2.52 (2.19)	0.30	3.58 (0.86)	0.93 (0.54)	0.31	3.54 (1.01)	0.80 (0.30)
rested	3.94 (1.80)	1.49 (0.46)	3.55 (2.74)	0.26	3.55 (0.92)	0.95 (0.56)	0.33	2.96 (1.20)	0.99 (0.31)
concentrated	3.77 (1.79)	1.37 (0.51)	3.37 (2.74)	0.34	3.95 (0.86)	0.77 (0.56)	0.37	2.85 (1.19)	0.91 (0.34)
active	3.92 (1.81)	1.41 (0.46)	3.43 (2.56)	0.34	3.66 (0.97)	0.85 (0.55)	0.42	2.95 (1.21)	0.94 (0.31)
interested	3.93 (1.82)	1.43 (0.52)	3.61 (2.62)	0.32	3.75 (1.06)	0.80 (0.59)	0.49	2.96 (1.22)	0.95 (0.35)
afraid	1.29 (0.93)	0.54 (0.57)	0.95 (1.49)	0.28	1.37 (0.56)	0.44 (0.57)	0.29	1.19 (0.62)	0.36 (0.38)
mad	1.46 (1.12)	0.83 (0.58)	1.73 (2.16)	0.19	1.42 (0.61)	0.55 (0.66)	0.24	1.30 (0.75)	0.55 (0.31)
unhappy	1.57 (1.20)	0.91 (0.59)	1.82 (2.29)	0.19	1.53 (0.67)	0.63 (0.66)	0.26	1.38 (0.80)	0.61 (0.39)
miserable	1.61 (1.23)	0.91 (0.59)	1.80 (2.25)	0.22	1.48 (0.68)	0.55 (0.64)	0.32	1.40 (0.82)	0.60 (0.40)
stressed	2.56 (1.73)	1.49 (0.48)	3.87 (2.82)	0.20	2.19 (0.94)	0.94 (0.59)	0.35	2.04 (1.16)	0.99 (0.32)
on edge	2.30 (1.70)	1.26 (0.63)	3.45 (3.03)	0.31	2.04 (0.99)	0.81 (0.62)	0.43	1.87 (1.13)	0.84 (0.42)
anxious	2.00 (1.49)	1.07 (0.60)	2.59 (2.69)	0.32	2.04 (0.99)	0.76 (0.62)	0.35	1.66 (0.99)	0.71 (0.40)
faint	1.88 (1.41)	1.06 (0.61)	2.17 (2.11)	0.26	1.68 (0.72)	0.68 (0.62)	0.29	1.59 (0.94)	0.71 (0.41)
exhausted	2.83 (1.79)	1.50 (0.48)	3.46 (3.47)	0.24	1.99 (0.83)	0.88 (0.59)	0.29	2.22 (1.19)	1.00 (0.32)
tired	3.04 (1.84)	1.44 (0.51)	3.02 (2.37)	0.32	2.19 (0.94)	0.85 (0.53)	0.41	2.36 (1.22)	0.96 (0.34)

SD, standard deviation, ISD, intraindividual standard deviation, MSSD, mean square successive differences, ICC, intraclass correlation, to compare the items the adolescent scores were transformed from 1–7 to 1–5 with the formula ((x-1)/6)*4 + 1.

To investigate the factor structure of momentary affect in adolescents, we conducted several two-level MCFAs with repeated measurements clustered within persons, identical to the analyses of Leonhardt et al. (2015). As we used the item set of Leonhardt et al. (2015), which was not constructed to represent true opposites on bipolar dimensions, we also used unipolar affect factors in our analyses. This decision was made based on previous work suggesting that, for example, positive and negative affect can be statistically independent (e.g., Watson and Tellegen, 1985) and comes with the advantage that the different factors are allowed to freely correlate. We tested three sets of models: a) a two-factor model with positive and negative affect; b) the pleasure-arousal model including factors *low-arousal*, *high-arousal*, *pleasure*, and *displeasure*; and c) a six-factor model with factors *tension*/*tiredness*, *alertness*/*calmness*, and *good*/*bad mood* to represent the three-dimensional model. To estimate the MCFAs, we used Mplus 8.2 (Muthén and Muthén, 2017) using maximum likelihood estimation with robust standard errors (MLRs). Missing data were handled with a full information maximum likelihood (FIML) approach, which is currently the approach and requires weaker assumptions to handle missing data than traditional approaches (Enders, 2010).

For model identification, the first factor loading of each latent variable was fixed to one. Model fit was evaluated with the χ^2^-test, the CFI, RMSEA, and the SRMR, with the latter being estimated separately for the within-person (SRMRw) and between-person (SRMRb) levels. To compare the fit of the different models, we conducted Satorra-Bentler scaled χ^2^-difference tests (Satorra and Bentler, 2001) and used Akaike’s Information Criterion (AIC) as a descriptive index. The alpha level for statistical significance was set to 0.05.

We calculated psychometric properties for the three models using McDonald’s Omega (Geldhof et al., 2014) to measure within- and between-person reliability. To correctly determine Omega, a scale must include at least three items. This is the case for all of our factors with only one exception. Unfortunately, the factor *calmness* in the six-factor model includes only two items. Thus, McDonald’s Omega cannot be calculated. We calculated a Spearman-Brown corrected correlation for *calmness* as the most appropriate reliability coefficient for a two-item scale (Eisinga et al., 2013).

## 3. Results

### 3.1. Descriptive and group comparisons

During the one-week assessment period, participants completed, on average, 34.5 (*SD* = 8.4; min = *6*, max = *62*) assessments and thus 5.0 (*SD* = 2.73) assessments a day. This translates to an overall compliance rate of 78.6% (*SD* = 17.5).

Table 1 reveals the mean as well as between- and within-person variance (i.e., ISD and MSSD) for all twenty single items of the six-factor affect model (Leonhardt et al., 2015). Columns 2 through 5 list the values of our adolescent dataset, and Columns 6 through 8 present descriptive data for children from the study by Leonhardt et al. (2015). Columns 10 and 11 provide the transformed mean and standard deviations of the adolescent sample. Starting with the adolescent sample, mean values ranged between 1.29 (*afraid*) and 5.21 (*cheerful*), given a possible item range of 1 to 7. In general, aggregating all negative items to one score revealed significantly lower values (*M* = 2.05; *SD* = 0.59) compared to the aggregated positive items (*M* = 4.53*; SD* = 0.80) (*t_(398)_ =* 28.99*, p* < 0.001). The correlation between the aggregated negative affect score and aggregated positive affect score was moderately negative (*r* = −0.38*, p* < 0.001), which is common for a healthy population. Standard deviations (*SDs*, values are shown in parentheses) ranged between 0.93 (*afraid*) and 1.84 (*tired*).

Regarding the mean levels and between-person standard deviations, we do not see evidence of pronounced ceiling or floor effects. In general, adolescents showed significantly higher means than children (except for *exhausted* (*t*_*(398)*_ = −2.26, *p* = 0.541) and *tired* (*t_(398)_* = −1.58, *p* = 0.835) values). Eight of the ten positive items yielded significant differences between the two samples, including *cheerful* (*t_(398)_* = 4.60, *p* = 0.004), *interested* (*t_(398)_* = 6.97, *p* < 0.001), *content* (*t_(398)_* = 4.81, *p* = 0.012), *good* (*t_(398)_* = 6.13, *p* < 0.001), *delighted* (*t_(398)_* = 3.77, *p* = 0.037), *active* (*t_(398)_* = 6.54, *p* < 0.001), *concentrated* (*t_(398)_* = 10.72, *p* <. 001), and *rested* (*t_(398)_* = 5.56, *p* < 0.001). These positive items were rated higher in adolescents. After Bonferroni-Holm correction only *concentrated*, *interested*, *active*, *good*, and *rested* could be classified as significant. In contrast, only one of the ten negative items (*anxious*, *t_(398)_* = 3.78, *p* = 0.030) showed significant differences between adolescents and children. Again, after Bonferroni-Holm correction also this difference could not be classified as significant.

From the within-person variation (i.e., *ISD*) in adolescents, we found values of between 0.54 (*afraid*) and 1.50 (*exhausted*) for the *ISDs* of the negative items and of between 1.06 (*cheerful*) and 1.49 (*rested*) for the positive items. The variation in affect over time within participants for the positive items was slightly greater than for the negative items. Comparing the mean of the *ISDs* across all ten positive items (*M* = 1.10, *SD* = 0.48, *min* = 0.18, *max* = 2.44) to the mean of the *ISDs* across all ten negative items (*M* = 1.25, *SD* = 0.39, *min* = 0.43, *max* = 2.46) revealed significantly higher values for the within-person variation in negative affect (*t_(398)_* = 7.34, *p* < 0.001). In addition, we found a strong correlation (*r* = 0.78, *p* < 0.001) between positive and negative within-person variation. This indicates that adolescents with high variability in positive affect also tend to have higher variability in negative affect. The *ISDs* of children and adolescents were quite similar. No differences between the samples regarding within-person variance showed significance (*t*-tests).

The mean square successive differences (Neumann et al., 1941; Ebner-Priemer et al., 2007), that is, instability, ranged from 1.66 (*cheerful*) to 3.87 (*stressed*). Aggregated positive *MSSDs* were slightly greater (*M* = 2.71, *SD* = 0.72, *min* = 0.03, *max* = 10.57) than aggregated negative MSSDs (*M* = 2.49; *SD* = 1.85, *min* = 0.42, *max* = 9.67) (*t_(398_* = 3.04, *p* = 0.003). This is in contrast to higher mean values of within-person variation in negative affect, as reported above. Again, we found a strong correlation (*r* = 0.84, *p* < 0.001) between positive and negative affective instability. This indicates that adolescents with high instability in positive affect also tend to have high instability in negative affect.

The *ICC* is defined by the ratio of between-person and total variance. For example, an *ICC* value of 0.30 indicates that 70% of the total variance can be attributed to within-person fluctuations (and 30% can be attributed to between-person differences). In our study, the *ICC*s ranged from 0.19 (*mad* and *unhappy*) to 0.39 (*cheerful*) (see Table 1), indicating that all items did show a substantial amount of within-person variance. Most *ICC*s were slightly lower in adolescents than in children. Specifically, the ICCs for the *stressed, interested, on edge, fantastic*, and *miserable* items were more than 10% lower in the adolescents than the *ICC*s reported by Leonhardt et al. (2015) for their sample of elementary school children. Only the *ICC*s for the *cheerful* (34% children; 39% adolescents) and *delighted* (32% children; 33% adolescents) measures were higher in adolescents, as shown in Table 1. To enable comparisons at the scale level to upcoming studies, Table 2 reports means, *ISD*s, *MSSD*s with their *SD*s, and *ICC*s for all factors of the three different models.

**Table 2:** Scale means, ISDs, MSSDs, and ICCs for the different models.

	Mean (SD)	Average ISD (SD)	MSSD (SD)	ICC
Two-Factor model				
Positive affect	4.50 (1.12)	0.77 (0.31)	0,59 (0.62)	0.46
Negative affect	2.05 (0.88)	0.62 (0.28)	0.77 (0.61)	0.35
Pleasure-arousal model				
Pleasure	4.77(1.19)	0.83 (0.34)	0.94 (0.77)	0.44
High Arousal	3.88 (1.45)	1.04 (0.36)	1.61 (1.14)	0.44
Displeasure	1.83 (0.87)	0.60 (0.30)	0.66 (0.73)	0.40
Low Arousal	2.59 (1.36)	1.06 (0.39)	1.48 (1.11)	0.33
Six-Factor Model				
Good mood	4.93 (1.27)	0.89 (0.39)	1.76 (1.41)	0.33
Calmness	4.38 (1.36)	1.06 (0.34)	1.11 (0.98)	0.42
Alertness	3.88 (1.49)	1.04 (0.34)	1.61 (1.14)	0.44
Bad mood	1.48 (0.86)	0.60 (0.39)	0.75 (1.00)	0.31
Tension	2.29 (1.21)	0.88 (0.34)	1.35 (1.04)	0.40
Tiredness	2.59 (1.36)	1.06 (0.39)	1.48 (1.11)	0.33

SD, standard deviation; ISD, intraindividual standard deviation; MSSD, mean square successive differences; ICC, intraclass correlation.

In summary, our first hypothesis that within-person variance reflects a major part of variation is confirmed in our data. This is in accordance with the child sample of Leonhardt et al. (2015).

### 3.2. Comparing factor structures across models

To investigate the factor structure of momentary affect, we conducted three MCFAs, the results of which are displayed in Table 3. Specifically, we tested three models, namely, the two-factor model (i.e., *positive* and *negative affect*), pleasure-arousal model (i.e., *pleasure*, *displeasure*, *low arousal*, and *high arousal*), and six-factor model (i.e., *good mood*, *bad mood*, *calmness*, *tension*, *alertness*, and *tiredness*). Overall, all models yielded adequate model fits. Specifically, the AIC showed decreasing values (i.e., a better model fit) with increasing model complexity from a two-factor to the full six-factor model, even though the AIC penalizes increasing complexity. In accordance with our second hypothesis, the six-factor model showed the best model fit. Supporting the findings of the AIC, the CFI also increased with model complexity from 0.79 (two-factor model) to 0.87 (pleasure-arousal model) to 0.89 (six-factor model). The RMSEA scored below the cutoff of 0.06 defined by Hu and Bentler (1999) and can therefore be interpreted as indicating a good model fit. The SRMR, which can be calculated for the between- and within-level tests separately, revealed values below the cutoff of 0.06 for the within-level scale only in the pleasure-arousal and six-factor models. At the between-person level, only the six-factor model reached a value close to the cutoff (0.07).

**Table 3:** Fit indices of all tested models.

Within-level and Between-level								
Model	No. of factors	Chi^2^	(df)	CFI	RMSEA	SRMR w	SRMR b	AIC
Two-factor model (positive affect, negative affect)	2	4416.24	(338)	0.792	0.043	0.067	0.117	405.998
Pleasure-arousal model (pleasure, displeasure, high arousal, low arousal)	4	2807.06	(328)	0.874	0.034	0.053	0.088	403.194
Six-factor model (good mood, bad mood, calmness, tension, alertness, tiredness)	6	2385.14	(310)	0.894	0.032	0.048	0.067	402.491

Chi^2^, Chi Square value of model fit; df, Degree of Freedom; CFI, Comparative Fit Index; RMSEA, Root Mean Square Error of Approximation; SRMRw, Standardized Root Mean Residual (within) SRMRb, Standardized Root Mean Residual (between); AIC, Akaike’s Information Criterion.

Testing the comparability of the within- and between-person structures with the Satorra-Bentler scaled χ^2^-difference test (Satorra and Bentler, 2001) revealed that all models with factor loadings constrained to be the same at the between- and within-person levels fit significantly worse than models without such constraints (two-factor model: Δχ^2^_(28)_ = 533.9, *p* < 0.05, pleasure-arousal model: Δχ^2^_(18)_ = 420.8, *p* < 0.05; and six-factor model: Δχ^2^_(14)_ = 48.5, *p* < 0.05). Nevertheless, the six-factor model showed the smallest difference, indicating that a comparable factor structure can explain between-person as well as within-person differences.

Table 4 shows the standardized factor loadings of all single items of the three affect models separately for the within- and between-person levels. For example, the item *stressed* had a factor loading of 0.33 for the *negative affect* factor of the two-factor model at the within level and a factor loading of 0.61 at the between level. When considering the factor loadings of the various models, the pleasure-arousal model (four-factor model) and six-factor model generally showed higher loadings than the two-factor model. The superiority of the six-factor model is further evidenced by fewer factor loadings with low values (e.g., no loadings of below 0.36 at the within-person level).

**Table 4:** Standardized factor loadings on the within- and between-person level for each tested model.

y	Model 1: Two-Factor Model			Model 2: Pleasure-Arousal Model			Model 3: Six-Factor Model		
	Factor Loading	W*	B*	Factor Loading	W*	B*	Factor Loading	W*	B*
cheerful	Positive affect	0.76	0.94	Pleasure	0.75	0.93	Good mood	0.76	0.94
content		0.74	0.96		0.75	0.96		0.75	0.96
good		0.78	0.98		0.78	0.98		0.78	0.99
delighted		0.73	0.96		0.73	0.96		0.73	0.96
fantastic		0.71	0.89		0.71	0.89		0.71	0.89
pleasant		0.63	0.90		0.63	0.90	Calmness	0.67	0.89
rested		0.30	0.64		0.29	0.63		0.36	0.67
concentrated		0.31	0.58	High arousal	0.57	0.94	Alertness	0.57	0.93
active		0.36	0.58		0.52	0.76		0.54	0.76
interested		0.45	0.64		0.61	0.95		0.59	0.96
afraid	Negative affect	0.37	0.75	Displeasure	0.40	0.76	Bad mood	0.40	0.77
mad		0.53	0.87		0.57	0.89		0.58	0.90
unhappy		0.66	0.94		0.70	0.95		0.72	0.97
miserable		0.67	0.92		0.69	0.92		0.70	0.93
stressed		0.33	0.61		0.30	0.59	Tension	0.43	0.68
on edge		0.30	0.72		0.32	0.70		0.39	0.88
anxious		0.40	0.81		0.43	0.79		0.49	0.93
faint		0.54	0.77	Low arousal	0.63	0.80	Tiredness	0.61	0.78
exhausted		0.45	0.58		0.66	0.85		0.68	0.86
tired		0.41	0.54		0.64	0.81		0.64	0.82

*W, within; B, between.

The more differentiated structure of the more complex models leads to an improvement in model fit and higher communalities. It seems that *displeasure* includes energetic and tension-related components; therefore, separating *displeasure* into two factors better fits the data. In contrast, the low-arousal factor of the pleasure-arousal model consists of the same items as the factor *tiredness* in the six-factor model, so we did not expect nor find major changes in factor loadings between the models. This could also be seen in the factors *high arousal* (pleasure-arousal model) and *alertness* (six-factor model). In summary, we confirm our second hypothesis, with the six-factor model showing the best model fit and descriptively higher factor loadings.

### 3.3. Correlations between latent factors

Table 5 displays the correlations of the within- and between-person level factors for the two-, four-, and six-factor models (Tables 5a,b,c, respectively). Factor correlations show the expected direction, ranging from *r*_=_ 0.50 (*tiredness*/*bad mood*) to *r* = 0.90 (*calmness*/*good mood*) at the within level and from *r* = 0.61 (*tiredness*/*bad mood*) to *r* = 0.99 (*calmness*/*good mood*) at the between level (see Table 5), and they show similar patterns in all three models.

**Table 5:** Correlations between factors at the within- and the between-person level.

a) Two-factor model							
		Between
		Negative affect	Positive affect				
Within	Negative affect		-0.41				
	Positive affect	-0.60					
b) Pleasure-arousal model							
		Between
		Pleasure	High arousal	Displeasure	Low arousal		
Within	Pleasure		0.63	-0.41	-0.43		
	High arousal	0.64		*-0.12*	-0.27		
	Displeasure	-0.58	-0.15		0.71		
	Low arousal	-0.47	-0.35	0.54			
c) Six-factor model							
		Between
		Good mood	Calmness	Alertness	Bad mood	Tension	Tiredness
Within	Good mood		0.99	0.62	-0.40	-0.30	-0.40
	Calmness	0.90		0.63	-0.54	-0.48	-0.58
	Alertness	0.64	0.74		*-0.13*	*-0.04*	-0.27
	Bad mood	-0.60	-0.50	-0.20		0.75	0.50
	Tension	-0.39	-0.45	0.12	0.71		0.58
	Tiredness	-0.45	-0.54	-0.35	0.61	0.76	

Above the main diagonal between correlations, and below the main diagnonal within correlations are reported. Values in italics are not significant.

In the two-factor model, the correlation of the *positive affect* and *negative affect* factors was *r* = −0.60 at the within level and *r* = −0.41 at the between level. The pleasure-arousal model yielded correlations of *r* = 0.64 (within) and *r* = 0.63 (between) for the positive factors *pleasure* and *high arousal*, whereas the negative factors *displeasure* and *low arousal* showed a correlation of *r* = 0.54 at the within level and *r* = 0.71 at the between level. In the six-factor model, *good mood* correlated with *alertness* at *r* = 0.64 within and at *r* = 0.62 between, whereas *good mood* and *calmness* correlated very high on both levels (*r* = 0.90 (within); *r* = 0.99 (between)). *Alertness* and *calmness* correlated within at *r* = 0.63 and between at *r* = 0.74. *Bad mood* correlated with *tiredness* at *r* = 0.50 (within) and *r* = 0.61 (between). *Tension* correlated with *bad mood* within at *r* = 0.71 and between at *r* = 0.75. *Tension* and *tiredness* correlated at *r* = 0.58 (within) and *r* = 0.76 (between).

### 3.4. Within- and between-person reliability

Table 6 displays McDonald’s Omega for all tested models and the Spearman-Brown corrected correlation coefficient for *calmness* in the six-factor model. At the between level, all models showed high coefficients. In the two-factor model, both factors reached a very high score (0.91 for *negative affect* and 0.94 for *positive affect*). In the pleasure-arousal model, the coefficients of the positive factors remained high (*high arousal* 0.92 and *pleasure* 0.93), whereas the coefficients of the negative factors decreased slightly to 0.86 for *low arousal* and 0.77 for *displeasure*. In the six-factor model, the five calculable factors yielded good reliabilities of between 0.88 (*tension*) and 0.98 (*good mood*).

**Table 6:** McDonald’s Omega for the different factor models.

		within			between		
		CI_−95_	McDonalds Omega	CI_+95_	CI_−95_	McDonalds Omega	CI_+95_
Two-Factor Model	Negative affect	0.68	0.72	0.75	0.88	0.91	0.94
	Positive affect	0.80	0.82	0.84	0.93	0.94	0.96
Pleasure-Arousal Model	Low arousal	0.65	0.68	0.71	0.76	0.86	0.95
	Displeasure	0.71	0.73	0.75	0.74	0.77	0.81
	High arousal	0.55	0.59	0.62	0.89	0.92	0.95
	Pleasure	0.85	0.87	0.88	0.92	0.93	0.93
Six-Factor Model	Bad mood	0.61	0.64	0.68	0.85	0.90	0.95
	Tension	0.35	0.41	0.46	0.85	0.88	0.92
	Tiredness	0.65	0.68	0.72	0.80	0.96	0.93
	Alertness	0.55	0.59	0.62	0.89	0.92	0.95
Good mood	0.84	0.86	0.88	0.97	0.98	0.98
Calmness^a^		0.39			0.74	

a In the six-factor model calmness consists of only 2 items. Therefore, Omega cannot be calculated and the Spearman-Brown corrected correlation is reported instead.

At the within-person level, Omega varied more across the different models. The two-factor model showed values of 0.72 (*negative affect*) and 0.82 (*positive affect*). In the pleasure-arousal model, the factor *displeasure* yielded a comparable Omega (0.73) as did negative affect in the two-factor model, whereas *low arousal* had a slightly lower value (0.68). In the positive direction, the factor *pleasure* reached a high value (0.87), while the arousal factor *low arousal* reached only a value of 0.59. In the six-factor model, except for *good mood* (0.86), lower Omegas were achieved, ranging from 0.41 (*tension*) to 0.68 (*tiredness*) for the remaining factors. *Calmness* showed a Spearman-Brown-corrected correlation coefficient of *r* = 0.74 for the between level and *r* = 0.39 for the within level.

Accordingly, our third hypothesis that repeated momentary measures of affective states add up to reliable affect factors could be fully confirmed at the between-person level and for at least four factors at the within-person level.

## 4. Discussion

Although hundreds of studies conducted over the last two decades have repeatedly assessed momentary daily life affect, psychometric information regarding model fit and reliability has rarely been provided. To the best of our knowledge, this is the first report of psychometrics conducted at the within-person level for affect scales in adolescents.

As hypothesized, our repeated momentary assessments revealed a considerable portion of within-person variance across all affect items. This is in line with the vast literature (Reichert et al., 2017; Giurgiu et al., 2020) showing consistently high levels of within-person variance in healthy and patient samples (Ebner-Priemer and Trull, 2012) and with the theoretical assumptions of affective dynamics, namely, that within-person dynamics are more important than between-person differences (Reichert et al., 2020; Santangelo et al., 2020; Koenig et al., 2021). In addition, reporting descriptive indices revealed no evidence for floor and ceiling effects. Comparing our adolescent sample with the child sample from Leonhardt et al. (2015) revealed no meaningful differences in mean values. Surprisingly, the instability indices of both groups were also comparable. This was unexpected, as, for example, Larson et al. (1980) reported more instability in adolescents, which is in line with theoretical assumptions regarding affective volatility in adolescence. Although Larson et al. (1980) compared their adolescent sample to an adult sample and not to children, this cannot explain the discrepant findings of our adolescent sample. We rather assume that missing heightened instability may partly be due to differences in study designs. While we assessed affect several times a day, Leonhardt assessed affect only once a day during school hours. As the time interval between assessments is especially important to assess dynamical processes (Ebner-Priemer and Sawitzki, 2007), studies of children using repeated measurements taken during the day and across different contexts would be necessary to allow for more interpretable comparisons.

Our second hypothesis of the six-factor model showing a better model fit than the two- and four-factor pleasure-arousal models is clearly confirmed by our sample of adolescents. Items tended to load more highly on the specific factors of the six-factor model than on the broader factors of the other models. Additionally, the six-factor model showed sufficient to good reliabilities on the between-person level, but insufficient reliabilities on the within-person level for some scales (e.g., Tension, Alertness, Bad Mood, and Calmness). In sum, and in accordance with the findings reported for a sample of children studied by Leonhardt et al. (2015), this model seems well suited to assess affective states in adolescent age groups.

Supporting our third hypothesis, the repeated momentary measures of affective states added up to reliable affect factors on the between- and within-person levels for all three models. Other than the indices of model fit, which increased with the complexity of the factor models, reliability tended to decrease with increasing complexity. This is not surprising, as increasing model complexity, that is, an increasing number of factors, led to fewer items per factor. However, reliability was still acceptable in the six-factor model.

Although we investigated the model fits in a reasonably large sample of adolescents for the first time, some limitations must be noted. First, the affect scales resulting from the different models included different numbers of items, rendering comparisons of reliabilities difficult to interpret. While short scales are warranted in e-diary research, having a minimum of three items per scale is necessary to estimate McDonald’s Omega. The within-person reliability also seems to have problems reaching sufficient levels on scales of three or fewer items.

Second, achieving a representative sample of experiences requires comprehensively covering various situations and contexts. However, prompting adolescents during school hours was not possible in our study. Accordingly, we prompted only in the afternoon after school and in the evening during the school week. Fortunately, we could take assessments at any time on weekends, which resulted in a mean of 34.5 assessments taken in 1 week. Compared to Leonhardt et al. (2015), who only assessed once a day, our sampling strategy should have captured a much greater variety of situations. As the study was conducted at the Central Institute of Mental Health in Mannheim, the sample could consist in parts with participants attracted to this address, but all participants were screened for acute diseases, mental disorders and others as exclusion criteria.

Third, including an additional adult comparison group with the exact same item set would have been desirable but was not possible. Generally, dedicated studies of the psychometric properties and model fit of repeatedly assessed affect in daily life in adults are also still missing and should be performed in future studies.

Fourth, while fundamental, reliability is not the only relevant psychometric property of scales used in experience sampling studies. In future studies, there should be an additional focus on other components of psychometric properties, such as validity, sensitivity to change, and reactivity in multilevel ambulatory assessment data.

## 5. Conclusions and recommendations

Psychometric properties and model fit should be reported by default in ambulatory assessment studies, as this is common in studies using validated questionnaires and as recommended in current guidelines (Trull and Ebner-Priemer, 2020).

The six-factor model presents a superior model fit relative to both the two-factor and pleasure-arousal models, while its factors show good reliability.

## Data availability statement

The data analyzed in this study is subject to the following licenses/restrictions: no permission for publication available. Requests to access these datasets should be directed to markus.reichert@rub.de.

## Ethics statement

The studies involving human participants were reviewed and approved by Medical Ethics Committee II of the Medical Faculty Mannheim at the Ruprecht-Karls-University in Heidelberg. Written informed consent to participate in this study was provided by the participants’ legal guardian/next of kin.

## Author contributions

MR, OB, AM-L, HT, and UE-P contributed to conception, design, and organization of the study. ML performed the statistical analysis and wrote the first draft of the manuscript. FS, PS, MR, LW, and UE-P consulted the statistical analysis and writing the first draft of the manuscript. All authors contributed to manuscript revision, read, and approved the submitted version.

## Funding

The URGENCY Study in which the data, used in this paper, was collected was founded by the Ministry of Science, Research and the Arts of the State of Baden-Wuerttemberg, Germany (MWK, grant 42-04 HV.MED(16)/16/1).

## Conflict of interest

AM-L has received consultant fees from the American Association for the Advancement of Science, Atheneum Partners, Blueprint Partnership, Boehringer Ingelheim, Daimler und Benz Stiftung, Elsevier, F. Hoffmann-La Roche, ICARE Schizophrenia, K. G. Jebsen Foundation, L.E.K. Consulting, Lundbeck International Foundation (LINF), R. Adamczak, Roche Pharma, Science Foundation, Sumitomo Dainippon Pharma, Synapsis Foundation – Alzheimer Research Switzerland, System Analytics, and has received lecture fees, including travel fees, from Boehringer Ingelheim, Fama Public Relations, Institut d’investigacions Biomèdiques August Pi i Sunyer (IDIBAPS), Janssen-Cilag, Klinikum Christophsbad, Göppingen, Lilly Deutschland, Luzerner Psychiatrie, LVR Klinikum Düsseldorf, LWL PsychiatrieVerbund Westfalen-Lippe, Otsuka Pharmaceuticals, Reunions i Ciencia S. L., the Spanish Society of Psychiatry, Südwestrundfunk Fernsehen, Stern TV and Vitos Klinikum Kurhessen. UE-P reports consultancy for Boehringer-Ingelheim.

The remaining authors declare that the research was conducted in the absence of any commercial or financial relationships that could be construed as a potential conflict of interest.

## Publisher’s note

All claims expressed in this article are solely those of the authors and do not necessarily represent those of their affiliated organizations, or those of the publisher, the editors and the reviewers. Any product that may be evaluated in this article, or claim that may be made by its manufacturer, is not guaranteed or endorsed by the publisher.
